# Design, Validity, and Reliability of a New Test, Based on an Inertial Measurement Unit System, for Measuring Cervical Posture and Motor Control in Children with Cerebral Palsy

**DOI:** 10.3390/diagnostics10090661

**Published:** 2020-09-01

**Authors:** Cristina Carmona-Pérez, Alberto Pérez-Ruiz, Juan L. Garrido-Castro, Francisco Torres Vidal, Sandra Alcaraz-Clariana, Lourdes García-Luque, Daiana Priscila Rodrigues-de-Souza, Francisco Alburquerque-Sendín

**Affiliations:** 1 Centro de Recuperación Neurológica de Córdoba (CEDANE), 14005 Córdoba, Spain; mcarperes@yahoo.es; 2 Doctoral Program in Biomedicine, University of Córdoba, 14004 Córdoba, Spain; m72alcls@uco.es (S.A.-C.); lgarcial05@hotmail.com (L.G.-L.); 3 Department of Nursing, Pharmacology and Physical Therapy, Faculty of Medicine and Nursing, University of Córdoba, 14004 Córdoba, Spain; n62perua@uco.es (A.P.-R.); falburquerque@uco.es (F.A.-S.); 4 Department of Computer Science and Numerical Analysis, Rabanales Campus, University of Córdoba, 14071 Córdoba, Spain; cc0juanl@uco.es (J.L.G.-C.); frantorresvidal@gmail.com (F.T.V.); 5 Maimonides Biomedical Research Institute of Cordoba (IMIBIC), 14004 Córdoba, Spain

**Keywords:** pediatric neurological disease, inertial sensors, control motor assessment

## Abstract

Objective: The aim of this study was to design and propose a new test based on inertial measurement unit (IMU) technology, for measuring cervical posture and motor control in children with cerebral palsy (CP) and to evaluate its validity and reliability. Methods: Twenty-four individuals with CP (4–14 years) and 24 gender- and age-matched controls were evaluated with a new test based on IMU technology to identify and measure any movement in the three spatial planes while the individual is seated watching a two-minute video. An ellipse was obtained encompassing 95% of the flexion/extension and rotation movements in the sagittal and transversal planes. The protocol was repeated on two occasions separated by 3 to 5 days. Construct and concurrent validity were assessed by determining the discriminant capacity of the new test and by identifying associations between functional measures and the new test outcomes. Relative reliability was determined using the intraclass correlation coefficient (ICC) for test–retest data. Absolute reliability was obtained by the standard error of measurement (SEM) and the Minimum Detectable Change at a 90% confidence level (MDC_90_). Results: The discriminant capacity of the area and both dimensions of the new test was high (Area Under the Curve ≈ 0.8), and consistent multiple regression models were identified to explain functional measures with new test results and sociodemographic data. A consistent trend of ICCs higher than 0.8 was identified for CP individuals. Finally, the SEM can be considered low in both groups, although the high variability among individuals determined some high MDC_90_ values, mainly in the CP group. Conclusions: The new test, based on IMU data, is valid and reliable for evaluating posture and motor control in children with CP.

## 1. Introduction

Cerebral palsy (CP) is a group of permanent disorders, attributed to a non-progressive damage during the fetal period or during the first years of life [1], that affects the normal development of movement and posture, causing disability and activity limitations [2]. Indeed, CP has been recognized as the most common origin of permanent physical disability in childhood, affecting between 1 to 3 per 1000 live births in Europe [3,4], and between 3 to 4 cases per 1000 among school-age children in the US [5]. The diagnosis of CP is mainly based on the clinical presentation of motor function and postural disorders [2].

Although cerebral palsy is associated with sensory deficits, cognitive, communication, and behavioral disorders, together with epilepsy and motor function disorders, represent the core symptoms [2], with spastic paresis being one of the most common forms of presentation [6,7]. This impairs the posture [8] and motor control [9], including the craniocervical region. The negative consequences of the alterations on posture and motor control of the craniocervical region in CP include, among others, an exacerbation of any feeding or swallowing disorder by the appearance of abnormal muscle tone and movement patterns [10]; a deterioration of the visual and vestibular senses, since the head is responsible for the directional orientation and its movements influence and are influenced by the information that both sensory faculties provide [11,12]; and, also associated to the alterations of the cranial senses, an increased risk of falls, because the maintenance of head stability has been identified as an important part of locomotor activity [13]. Commonly, the assessment tools used for motor disorders in CP are based on the observation of individual functional abilities [14,15,16]; however, these measures are considered subjective [11,17]. Indeed, other specific approaches are also necessary in clinical settings and in research [11,18] based on the analysis of movement and posture [19].

One of the most used assessment tools to study human position and movements are the inertial measurement units (IMUs), due to their portability, ease of application, the high quality of obtained data, and low energy consumption [20]. Nevertheless, as occurs with any new assessment tool, all the IMU applications should be completely described, reproducible, and validated [20,21,22] to establish clinical meaningfulness and predictive importance [19,23,24]. Thus, the IMUs have been successfully applied in the study of specific features of neurological diseases, such as range of motion in stroke [25], Parkinsonian tremor [26,27], or balance in multiple sclerosis [28,29]. In neurological pediatrics, and specifically in lower limbs and gait analysis, the validity and reliability of IMUs has been demonstrated [30,31]. To date, few studies have evaluated the craniocervical features of IMUs applications in CP, although those available have obtained good validity and reliability results [17,32].

Thus, the aim of this study was to design and propose a new test based on IMU technology for measuring cervical posture and motor control in children with CP. Further, the aim included the determination of the metric features, in terms of validity and reliability, of the new test when applied in children with CP and healthy controls. We hypothesized that the new test would display good validity and good test–retest reliability in children with CP and healthy controls, which would allow its implementation in clinical setting.

## 2. Materials and Methods

### 2.1. Individuals

The design of the new test was developmental and descriptive. Subsequently, a clinical measurement study assessing construct and content validity, and test–retest reliability was performed in a two-stage repeated measures design, which took place from January 2018 to March 2020. Patients with CP were recruited from the private Neurological Recovery Center of Córdoba (CEDANE), in Spain, using non-probabilistic sampling of consecutive cases. The inclusion criteria were children from 4 to 14 years old diagnosed with CP; sufficient cognitive and behavioral skills for understanding tasks and following simple instructions; Gross Motor Function Classification System (GMFCS) levels I–IV; a level of 3 or higher in the Manual Muscle Test of cervical muscles [33,34]; clinically stable. The exclusion criteria were aggressive/self-injurious behavior; uncontrolled epilepsy/seizures (stable epilepsy with medication for more than 12 weeks); involuntary/uncontrollable head or trunk movement that prevent the application of the study protocol; orthopedic surgery at least 1 year before the evaluation; administration of botulinum toxin at least 6 months before the evaluation; treatment with anti-spasticity drugs at the time of the assessments; bone deformities, contractures or tactile hypersensitivity that do not allow the body alignment; severe visual limitations; suffering from pain; participation in other biomedical research.

A group of control individuals, with no neurological or other impairments, matched for gender and age (±2 years), were also selected. They were recruited via the researchers’ personal contacts and from the University of Córdoba (Spain).

The Body Mass Index was determined for all subjects, according to the Z-score of the United States Centers for Disease Control and Prevention [35]. The CP individuals were classified regarding the type of movement disorders (e.g., spastic, dyskinetic, ataxic, mixed) [36,37]. Further, the spasticity was assessed with the modified Ashworth scale for cervical flexor, extensor, and sternocleidomastoid muscles. This scale has shown acceptable reliability in CP [38], and it is scored as follows. 0: No increase in muscle tone. 1: Slight increase in muscle tone, manifested by minimal resistance at the end of the range of motion in flexion or extension. 1+: Slight increase in muscle tone, manifested by minimal resistance in less than half of the ROM. 2: More marked increase in muscle tone, but affected part(s) can be easily moved. 3: Considerable increase in muscle tone, passive movement difficult. 4: Affected part(s) rigid in flexion or extension [39].

All parents or caregivers of study individuals gave their informed consent prior to participating in the study. This study protocol was approved by the Ethics Committee of Reina Sofía University Hospital (reference 3680-17, 6 November 2017 approved).

The sample size required to test the concurrent validity between the outcomes of the new test and functional scores was based on a bilateral Pearson’s correlation coefficient, assuming an expected correlation of *r* ≥ 0.60, a level of significance of 5%, and 90% power. Thus, we determined that at least 21 individuals were necessary in the CP group. In addition, based on previous studies [32,40,41], and considering an intraclass correlation coefficient (ICC) of 0.8, an accuracy of 0.23, and a level of significance of 5%, the estimated sample should consist of at least 22 individuals (Tamaño de la muestra 1.1^®^ software (Pontificia Universidad Javeriana, Bogotá, Colombia). Due to the follow-up period, 10% data loss was expected, and 24 individuals were assessed in each group.

### 2.2. Cervical Motor Control Test Development and Application

To develop the new test, a literature research and two proofs of concept were performed to improve the content validity of the new test [42]. The first proof of concept consisted of the assessment of the protocol feasibility in two children, one of which was a CP patient and one of which was a healthy child. Both assessments were recorded and submitted to a focus group of four experienced professionals from diverse healthy science backgrounds (pediatric physical therapy (Cristina Carmona-Pérez), musculoskeletal physical therapy (Daiana Priscila Rodrigues-de-Souza), research methodology (F.A.-S.), and biomechanical engineering (Juan L. Garrido-Castro). The size of the group was restricted to facilitate discussion. The second proof of concept aimed to test the IMU protocol regarding the collection, extraction, transfer, and analysis of data. One more assessment was performed in a healthy child, and all data were analyzed by the biomechanics engineer (Juan L. Garrido-Castro) who participated in the first proof of concept and a computing engineer (Francisco Torres Vidal). The recorded raw data based on flexion/extension and rotation angles were plotted and following a covariance calculation, an eigenvalues and chi-square distribution were used to define the ellipse, which bounds 95% of the raw data. On the basis of the focus group discussion and the second proof of concept, the preliminary test was refined, resulting in the final version that was used in this study, as described below.

**Figure 1:**
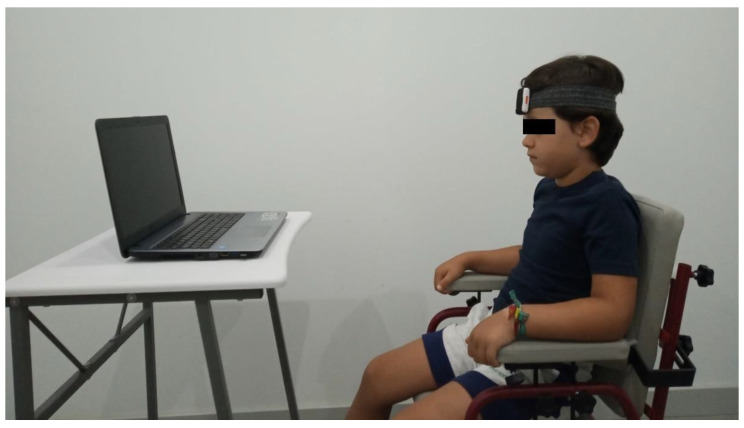
Inertial measurement unit (IMU) test application: sensor, subject, and laptop location.

The general recommendations for assessments in children were applied. Thus, relatives or caregivers who were functionally involved and part of the daily relationship (relatives/caregiver/child) were included in the procedures [43,44]. The evaluation was performed in a quiet room, in which the assessors and relatives/caregiver were present, alongside the study individual. No other people were present. The individual was seated on a non-swivel chair in a standardized manner. The chair was adapted in length, width, and height to the anthropometric characteristics of each child, and straps and other orthopedic elements were used when necessary to secure and reproduce the body alignment according to the usual posture of the child. Moreover, the child and the caregivers were asked to report any discomfort while seated. A flexible and adjustable strap was attached to the head to support an IMU Shimmer3 ^®^ sensor placed on the individual’s forehead. The sensor captured the orientation in the three planes of movement at 50 Hz, and it was connected to an android mobile phone using iUCOTrack © software (iSAB, Córdoba, Spain) [45,46] for the acquisition and processing of the raw data. To calibrate the IMU, at the beginning of the test, the child was instructed to keep the eyes fixed on the monitor of a laptop (17″ screen), placed 1 m in front of the child (Figure 1). The assessor observed that there were no deviations from this position, which was determined as the initial static position, from which the differences in the three planes of movement were collected. Next, a two-minute video, chosen by the child among various cartoons and music videos, was shown to the individual. Specific instructions were given to the individual to perform the test, as follows: “You will be watching the video for 2 min, and you have to be as still as possible”. The individuals were also instructed to avoid shoulder or thoracic movements. They were asked whether any pain appeared during the evaluation. In the event of pain, the procedures were interrupted. All tests were performed by an experienced physiotherapist (C.C-P.), with over 15 years’ experience working with CP patients.

For reliability purposes, data were collected on two different occasions, 3 to 5 days apart, applying the same protocol. On the second day, the assessor was blinded to all previous data [20].

**Figure 2:**
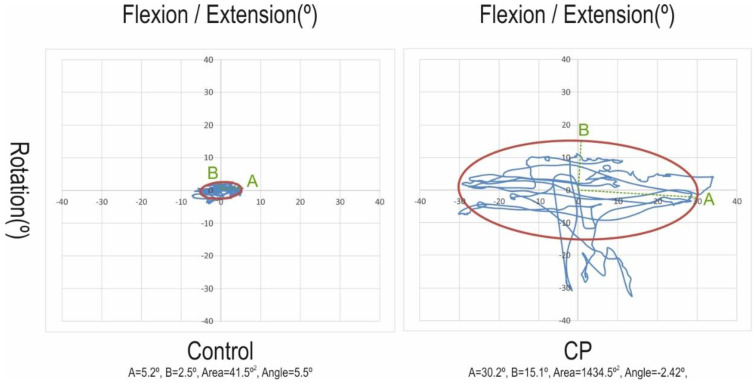
Ellipse encompassing 95% of the flexion/extension and rotation displacements for control and cerebral palsy (CP) individuals.

The first 10 s of the test were removed for the data processing of each test. Subsequently, a Butterworth low-pass frequency filter of 10 Hz was applied. The variables obtained can be divided into two types. The first type is the characterization of the registered movements, considering angle (in relation to the initial position), angular velocity, angular acceleration in the three planes, and total angle distance covered during the test. The Root Mean Square (RMS) of the angular displacement, as well as its velocity and acceleration, were analyzed in each plane and summarized. Besides the movement per plane (sagittal-flexion/extension, transverse-rotation, coronal-lateral bending), a mean angle, as the mean of the three orientation angles, was calculated. The second type includes the components of an ellipse obtained by calculating the eigenvalues of the covariance matrix between the flexion/extension and rotation angle that covers 95% of the data (Figure 2). The area of the ellipse, the angle (direction of the principal axis), the size of its flexion/extension displacements on the ellipse (A-dimension), and measures related to rotation displacements (B-dimension) were the dependent variables.

### 2.3. Funcional Assessment

To evaluate the functional state in CP individuals, two approaches were applied. First, for assessing the execution of motor sills, the Gross Motor Function Measure (GMFM-88) was applied [47]. The GMFM-88 consists of 88 items grouped into five dimensions: A (lying and rolling, composed of 17 items), B (sitting, composed of 20 items), C (crawling and kneeling, composed of 14 items), D (standing, composed of 13 items), and E (walking, running, and jumping, composed of 24 items). Each item is scored on a Likert scale (4 points for each item). A percentage score is calculated for each dimension. Overall scores can also be calculated as the mean of the five dimension scores [48]. The reliability, validity, and responsiveness of the GMFM-88 scores are documented for children with cerebral palsy [49,50]. The Spanish version of the GMFM-88, which has shown excellent reliability, for inter-assessor (ICC = 0.998–1; 95% confidence interval (95% CI) = 0.986–1), intra-assessor (ICC = 0.999–1; 95% CI = 0.999–1) and test–retest (ICC = 0.991–1; 95% CI = 0.971–1), both by dimensions and total score, was used in this study [51,52].

Subsequently, the Pediatric Evaluation of Disability Inventory (PEDI) was applied to assess the performance of activities relevant to daily function in both activity and participation domains. The PEDI evaluates 197 specific tasks divided into three domains: Self-Care (composed of 73 items), Mobility (composed of 59 items), and Social Function (composed of 65 items). All the items in each domain are scored as follows: a score of 1 indicates capability of performing the described task independently; a score of 0 indicates inability to perform a task or requiring assistance. The sum of scores on each item for each domain is calculated, and finally, the global score is obtained by the sum of the three domain scores. The PEDI has shown good psychometric properties [50,53]. The Spanish version of the PEDI, which has shown high internal consistency (Cronbach’s alpha = 0.930; 95% CI = 0.890–0.950) and excellent test–retest reliability (ICC = 0.980, 95% CI = 0.982–0.993 for the Self-Care domain; ICC = 0.990, 95% CI = 0.990–0.996 for the Mobility domain; ICC = 0.980, 95% CI = 0.972–0.990 for the Social Function), was used in this study [54].

### 2.4. Statistical Analysis

Frequencies, percentages, means, standard deviations, and 95% CI were used for describing quantitative and qualitative variables. The normality of the quantitative variables was tested and confirmed using the Shapiro–Wilk test (*p* > 0.05).

#### 2.4.1. Validity Analysis

Construct validity was determined in two different manners by comparing the outcomes of the new test obtained during the assessment on the first day between CP individuals and the control group. First, the differences in outcome data between both groups were identified using unpaired t-tests. Secondly, also, a Receiver Operating Characteristic (ROC) curve was applied to assess whether the new test data were able to discriminate between CP individuals and controls. The Area Under the Curve (AUC) and the statistical significance of the ROC curve were reported. Furthermore, the same analyses were performed between the non-wheelchair user individuals (GMFS I–II) and wheelchair user individuals (GMFS III–IV) of the CP group. In this case, the Mann–Whitney U test was applied to compare both subgroups of CP individuals.

To assess concurrent validity, in the CP group, the Pearson’s correlation coefficient (*r*) was applied among sociodemographic data and outcomes obtained during the first day assessment, GMFM and PEDI domains, and total scores. Correlation coefficient values were considered as weak (0.0 to 0.3), moderate (0.4 to 0.6), or strong (0.7 to 1.0) [55]. Furthermore, the new test results, along with the sociodemographic data, were included in a stepwise multiple regression model to estimate whether these variables can explain the variance of the functional state (GMFM-88 total score and PEDI total score) of the individuals. Multicollinearity and shared variance were assessed, defined as *r* > 0.80 between the variables. A *p*-value of 0.05 was set as the significance criterion of the critical F value for entry into the regression equation. The changes in R^2^ were reported after each step of the model.

#### 2.4.2. Reliability Analysis

The relative test–retest reliability of new test outcomes, based on the assessments performed on different days, was determined by calculating ICC for test–retest reliability (ICC2,1) in each group [56]. ICC values below 0.20 were considered poor, from 0.21 to 0.40 were considered reasonable, from 0.41 to 0.60 were moderate, from 0.61 to 0.80 were good, and from 0.81 to 1.00 were very good [41]. Paired t-tests were also used to analyze the differences between outcome data between both days.

The absolute reliability was assessed using the standardized error of measurement (SEM), which was calculated as SEM = SD_pooled_ × (1 − ICC), where SD_pooled_ is the standard deviation of the scores, and the minimum detectable change (MDC) at 90% confidence level was calculated as SDC = 1.96 × √2 × 1.64.

The SEM provides a value for the random measurement error in the same unit as the measurement itself, which quantifies the variability within the individual and reflects the amount of measurement error among assessments [57,58]. The MDC is an estimate of the smallest amount of change that can be objectively detected as a true change outside the measurement error when separate measures are performed [57,59]. Furthermore, the MDC_90_ was used to determine the effectiveness of interventions [40].

All hypothesis tests were considered significant if *p* was less than 0.05, because the validity and reliability analyses were based on independent *a priori* hypotheses [60]. The data were managed and analyzed with IBM-SPSS^®^, version 25.

## 3. Results

The present study included 24 children in the CP group and 24 children in the control group. The mean age of the sample was 9.0 ± 3.3 years. In total, 62.5% of the CP individuals were non-wheelchair users (GMFCS levels I–II), whereas 37.5% were considered wheelchair users (GMFCS levels III–IV). Regarding the type of movement disorders in the CP group, 83.3% of the individuals were spastic, while 12.5% were dyskinetic, and 4.2% were classified as mixed. None of the individuals presented a score of more than 2 in the modified Ashworth scale in any cervical muscle group, and none of the individuals suffered from pain while undergoing the evaluations. Table 1 displays detailed descriptive data. 

### 3.1. Construct Validity

Considering the characterization of the movement variables, the angular movement in each plane and the mean was significantly greater in the CP group. Although the remaining variables were also greater in the CP group, statistical differences were not observed, which was probably due to the high variability of data. The same trend was identified in the ellipse variables, where all means were higher in the CP group, although the statistical significance was exclusively observed for the Area, A-dimension, and B-dimension (Table 2).

The highest discrimination between case and controls was shown by B-dimension and the area of the ellipse (Area Under the Curve (AUC) > 0.8). The A-dimension also achieved statistical significance, with Distance and Angle of the ellipse without discriminant capacity (AUC ≈ 0.5). The Receiver Operating Characteristic (ROC) curve of the summarized variables of the characterization of movements showed that only the mean angle was able to discriminate between CP and control individuals (AUC = 0.746; *p* < 0.05) (Supplementary Materials Figure S1).

None of the variables concerning characterization of the movement were able to discriminate non-wheelchair users and wheelchair users, whereas the AUCs of the Area and A-dimension were statistically significant (AUC = 0.708 and 0.808, respectively) (Supplementary Materials Figure S2). The only variable that revealed statistical differences between both subgroups of CP individuals were the flexion-extension angle and the A-dimension of the ellipse.

### 3.2. Concurrent Validity

A consistent trend of moderate and strong associations between the functional scores and the results of the new test was detected. Thus, flexion-extension angle, mean angle, lateral velocity, and angle and Dimension-A of the ellipse were correlated to all the GMFM-88 dimensions and total score. On the contrary, flexion-extension acceleration, rotational acceleration, and Dimension-B of the ellipse were not associated to any GMFM-88 result (Table 3).

For PEDI, all the results of the new tests were correlated with the PEDI Self-Care domain, and with all PEDI domains and total score in many cases. Furthermore, the Self-Care domain was correlated with all the new test outcomes. This pattern included some strong correlation coefficients involving flexion-extension angle root mean square error (RMSE) and Dimension-A of the ellipse and PEDI scores (Table 3).

Table 4 summarizes the hierarchical regression analysis for both functional total scores. For the GMFM-88 total score, the model achieved a 63.8% explanation of variance including the flexion-extension angle RMSE and two sociodemographic variables. Thus, the regression coefficients showed that lower flexion-extension movement (explaining 43%), age, and higher height were associated with higher GMFM-88 values. In this case, no variable of the ellipse was included in the model.

When the PEDI total score was considered the dependent variable, a model including two variables of the ellipse, the rotational acceleration and age, explained 83.3% of the variance. Thus, the regression coefficients showed that a more reduced area (explaining 56%) and distance of the ellipse, age, and higher rotation acceleration were associated with higher PEDI values.

### 3.3. Test–Retest Reliability

In general, the reliability analysis determined that the ICCs were higher in the CP group than in the control group. Specifically, the ICCs of the CP group ranged from 0.82 to 0.94, except for the Angle and A-dimension of the ellipse, which were lower. For the control group, the values were more variable, ranging from 0.51 to 0.94. The highest values were showed by angle RMSE and area and A-dimension of the ellipse (ICC > 0.9). The 95% CI of all outcomes and both groups showed a trend of (upper limit: ICC + 0.3, lower limit: ICC − 0.3), with the exceptions of lateral and rotational accelerations RMSE, and distance and angle of the ellipse, of the control group, which showed a higher amplitude of the 95% CI. No differences were detected between data obtained on both days for any outcomes and for both groups (*p* > 0.05).

The absolute reliability data were variable, with the SEM of angular movements below 3.2°, and the velocity equal or below 4.0°/s for both groups in all cases. For the ellipse variables, the SEM were higher for the CP group. This trend was also observed for the MDC_90_ with higher values for the CP group, which was associated with the variability of the results among individuals. All reliability results were included in Table 5.

## 4. Discussion

The application of a new test based on IMU technology for assessing the motor control of the craniocervical region in CP children has been demonstrated as being both valid and reliable. Furthermore, no individuals suffered from pain or any other complaint during the execution of the test, confirming the hypotheses and increasing the possibilities of applying this test in clinical settings. This new test was able to discriminate between CP individuals and controls, with better results for some of the ellipse variables. Furthermore, the new test variables were moderate to strongly associated with functional measures, and these total scores were partially explained by the combination of movement and ellipse variables of the new test and sociodemographic measures such as age and height. The relative reliability was very good, and the SEM for both groups were acceptably low. Some MDC_90_ could be high for test–retest comparisons among CP individuals, and caution is recommended when it is applied as a parameter to detect the effects of therapeutic interventions.

Previous research has determined the clinical areas of the application of IMUs in CP individuals; concretely, these have been used for the following: (1) Objective diagnosis of motor disorders; (2) Proprioceptive rehabilitation based on visual-motor feedback; and (3) Functional compensation by means of an inertial person–machine interface [17]. Furthermore, instrumented methods, and specifically IMUs, may lead to a better understanding of the pathophysiological aspects of CP and help guide clinical decision making (e.g., quantifying deficits and determining progress in time) [23,61]. The current study adds a new assessment tool for the diagnosis and assessment of craniocervical posture and motor control impairments in CP children. This is supported by the capacity to discriminate between cases and controls, which has reached its maximum capacity in the area and A and B-dimensions of the ellipse (AUC ≈ 0.8).

Regarding applicability, the design of the test and the absence of complaints reported by individuals during the assessment demonstrates high feasibility and safety of the protocol, which can be used outside the laboratory. This characteristic is an important feature of any new instrumented propose for motor function assessment in cerebral palsy [23]. In this sense, since motivation is a part of any attendance procedure in CP success, the ellipse obtained by the covariance matrix, its size and form, affords a form of visual feedback, which is easy to understand for clinicians, patients, and caregivers, as it has been reported for other applications of IMUs [62,63].

The link between postural control and functionality is well known [8]. The current results of the new test showed a stable pattern of association with functional parameters in CP individuals, with this pattern being more evident for the performance of activities relevant to daily function in both activity and participation domains, assessed with PEDI, rather than the capacity of execution of motor skills, assessed by the GMFM-88 [64]. Thus, the amount of the flexion-extension movement was inversely proportional to the execution of motor function, which can be explained by the need of a constant motor control of the head against gravity to maintain the posture [32]. Although a specific explanation of the relationship between age and gross motor function is beyond the objective of this paper, the different demands of the GMFM-88 according to patient age could justify this association. Interestingly, none of the variables of the ellipse were included in the gross motor function model, which may be due to a high capacity of the ellipse to detect fine motor control, as these features were more common in the tasks assessed with PEDI. Indeed, the area of the ellipse was able to explain over 50% of the variance of the functional abilities assessed with PEDI. Thus, a higher combination of movements on the sagittal and rotational planes were strongly associated with poorer functional tasks. Furthermore, when a bivariant approach was observed, the anterior–posterior dimension of the ellipse was strongly related to all PEDI results. Finally, the specific need for fast motor control adjustments when the patient is watching the computer screen, which is also necessary in many daily tasks, could explain the positive relation between function and rotational accelerations [65].

This pattern of association is highly relevant, since the functional questionnaires used in the current study evaluate the function of the whole body, which highlights the hegemony of the head in many tasks. Indeed, motor control of the head is relevant for balance [13], including static postures [66,67]. Moreover, the poor motor coordination of the head in CP individuals can be a cause of difficulties in the planning and executing precise movements [17].

Most of the papers that have studied the validity of IMU applications in CP are focused on lower limb movements and gait [68,69,70,71], which limits their application to individuals with preserved gait. Our approach extends the validity assessment of motor control to subjects with GMFCS III and IV in a consistent approach. Furthermore, a lower validity of IMU applications concerning the rotational plane has been reported for determining the craniocervical range of motion in CP [32] and healthy individuals [65], which was not detected in the current study.

It has been reported that one of the strong points of IMUs is the high reliability trend of the assessments and the low errors of measurement: in general, between 2° and 5° [20,72]. The relative test–retest analysis of the new test was, for almost all the variables, very good in the case of CP individuals, and good to very good in controls. In addition, the SEM, as a measure of absolute reliability, can be considered acceptable, since all angular values were below 4° in both groups. This can be considered a reference point, since the other characteristics of the movement, such as velocity or acceleration, are derived from the angular movements. Nevertheless, the MDC_90_ of CP individuals were higher than the MDC_90_ of controls—in some cases by more than 100%. In line with previous research findings involving IMUs [32], high MDC_90_ hampers their applicability to detect an effect when a therapeutic intervention is applied in research or clinical settings. The pattern of high relative reliability with less absolute reliability, mainly in CP individuals, is probably due to the high variability of the movement evaluations [73]. In fact, ICC increases with higher between-individual variance [59]. The heterogeneity of the level of patients’ affectation, the variation of spasticity states [74], and the training effect between both assessments [75] could explain part of the variability. Further research should identify whether more homogeneous subgroups show lower MDC.

Despite the promising results of the current study, some limitations were identified. First, the new test exclusively evaluates the craniocervical region, although all body regions can be affected by losses of motor function in CP patients. Nevertheless, limbs are usually more affected than the craniocervical region in children with severe CP [76], which could reduce the feasibility of the new assessment approaches. Thus, the craniocervical region can be identified as a good reference for the performance of motor control evaluations, as proposed in other research [17,19]. Second, as previously commented, the metric features of any assessment tool are population specific. Indeed, the applicability of the new test is limited to similar samples. More research is necessary to apply these results to other age ranges or populations with specific levels of functional impairment. Third, the current study only assessed the craniocervical motor control in a specific, simple, and controlled setting, which cannot be extrapolated to more complex tasks and different conditions [20]. In summary, further research is necessary, considering more complex assessment protocols and different populations, with the aim of standardizing technical procedures and obtaining normative data [65].

## 5. Conclusions

The new test for measuring cervical posture and motor control, based on IMU technology, is valid and reliable for CP children. However, caution is recommended when applying this test to detect the effects of an intervention. Its application in clinical settings can be considered feasible, providing a visual feedback that is easy to understand.

## Figures and Tables

**Table 1:** Demographic and clinical characteristics of the individuals.

	CP Group (*n* = 24)	Control Group (*n* = 24)	*p*-Value
Age (years)	9.1 (3.0)	8.8 (3.2)	0.720
Sex (women/men)	15/9	15/9	
Weight (kg)	28.5 (12.9)	33.2 (14.0)	0.250
Height (m)	1.33 (0.22)	1.35 (0.23)	0.736
BMI (Z-score)	−0.15 (1.19)	0.08 (1.30)	0.171
GMFCS level (frequency)	I: 11; II: 4; III: 1; IV: 8	-	-
Type of motor disorder (frequency)	Spastic: 20; Dyskinetic: 3; Ataxic: 0; Mixed: 1	-	-
GMFM-88		-	-
Dimension A	81.7 (24.3)	-	-
Dimension B	73.5 (32.9)		
Dimension C	62.8 (39.4)		
Dimension D	54.2 (39.4)		
Dimension E	46.1 (38.3)	-	-
Total score	63.6 (33.7)	-	-
PEDI		-	-
Self-Care	24.1 (13.52)	-	-
Mobility	20.1 (12.8)	-	-
Social Function	18.8 (7.3)	-	-
Total Score	63.0 (32.1)	-	-

Quantitative data are expressed as mean (standard deviation). Abbreviations: CP, cerebral palsy; GMFCS, Gross motor function classification system; GMFM-88, Gross Motor Function Measure; PEDI, Pediatric Evaluation of Disability Inventory; BMI, body mass index; “-” means that no value is necessary.

**Table 2:** Comparison of the new test results obtained on the first day between the CP and control groups.

	CP Group (*n* = 24)	Control Group (*n* = 24)	Mean Difference (95% CI)	*p*-Value
Movement characteristics				
Flexion-extension angle (°)	8.59 (8.69)	3.93 (2.88)	−4.66 (−8.56; −0.75)	0.021
Rotational angle (°)	10.29 (11.30)	2.57 (2.53)	−7.72 (−12.70; −2.75)	0.004
Lateral angle (°)	7.16 (6.01)	2.59 (2.36)	−4.57 (−7.32; −1.83)	0.002
Mean angle (°)	8.68 (7.78)	3.03 (2.20)	−5.65 (−9.11; −2.19)	0.002
Flexion-extension velocity (°/s)	8.77 (9.92)	4.85 (3.97)	−3.92 (−8.45; 0.61)	0.087
Rotational velocity (°/s)	10.35 (14.64)	4.24 (5.75)	−6.11 (−12.79; 0.57)	0.071
Lateral velocity (°/s)	8.22 (9.25)	4.72 (4.22)	−3.49 (−7.79; 0.81)	0.107
Mean velocity (°/s)	9.11 (11.13)	4.61 (4.56)	−4.51 (−9.61; 0.59)	0.081
Flexion-extension acceleration (°/s^2^)	128.75 (157.13)	107.92 (75.53)	−20.82 (−94.40; 52.75)	0.568
Rotational acceleration (°/s^2^)	128.54 (175.01)	91.48 (62.83)	−37.06 (−116.23; 42.11)	0.345
Lateral acceleration (°/s^2^)	142.52 (159.44)	121.98 (92.79)	−20.53 (−97.85; 56.78)	0.593
Mean acceleration (°/s^2^)	133.27 (162.05)	107.13 (75.02)	−26.14 (−101.60; 49.32)	0.485
Ellipse variables				
Distance (°/s)	11.61 (13.75)	6.43 (4.86)	−5.18 (−11.39; 1.04)	0.099
Area (°^2^)	857.18 (1374.52)	91.33 (206.35)	−765.85 (−1364.98; −166.72)	0.015
Angle (°)	7.01 (27.01)	0.64 (21.80)	−6.38 (−20.59; 7.83)	0.371
A-dimension (°)	12.34 (12.24)	4.59 (3.32)	−7.75 (−13.18; −2.31)	0.007
B-dimension (°)	13.25 (13.96)	3.65 (5.11)	−9.60 (−15.92; −3.27)	0.004

Data are expressed as mean (standard deviation). Abbreviations: CP, cerebral palsy; CI, confidence interval.

**Table 3:** Correlations between sociodemographic, new test outcomes, and functional scores in the CP group (*n* = 24).

	Dimension A	Dimension B	Dimension C	Dimension D	Dimension E	GMFM-88 Total Score	Self-Care	Mobility	Social Function	PEDI Total Score
Age	n.s.	n.s.	n.s.	n.s.	n.s.	n.s.	n.s.	n.s.	n.s.	n.s.
Weight	n.s.	n.s.	n.s.	n.s.	n.s.	n.s.	n.s.	n.s.	n.s.	n.s.
Height	n.s.	n.s.	n.s.	n.s.	n.s.	n.s.	n.s.	n.s.	n.s.	n.s.
BMI	n.s.	n.s.	n.s.	n.s.	n.s.	n.s.	0.414; 0.048	n.s.	0.596; 0.003	0.442; 0.035
Flexion-extension angle	−0.738; <0.001	−0.677; <0.001	−0.615; 0.002	−0.589; 0.003	−0.626; 0.001	−0.663; 0.001	−0.723; <0.001	−0.702; <0.001	−0.590; 0.003	−0.715; <0.001
Rotational angle	−0.446; 0.033	n.s.	n.s.	n.s.	−0.432; 0.040	n.s.	−0.575; 0.004	−0.472; 0.023	−0.583; 0.004	−0.561; 0.005
Lateral angle	n.s.	n.s.	n.s.	n.s.	−0.465.026	n.s.	−0.476; 0.022	−0.419; 0.046	−0.463; 0.026	−0.472; 0.026
Mean angle	−0.569; 0.005	−0.523; 0.010	−0.478; 0.021	−0.451; 0.031	−0.562; 0.005	−0.530; 0.009	−0.670; <0.001	−0.597; 0.003	−0.622; 0.002	−0.660; 0.001
Flexion-extension velocity	−0.516; 0.012	−0.447; 0.032	−0.415; 0.049	−0.348	−0.434; 0.034	−0.439; 0.036	−0.574; 0.004	−0.479; 0.021	−0.516; 0.012	−0.549; 0.007
Rotational velocity	n.s.	n.s.	n.s.	n.s.	n.s.	n.s.	−0.500	n.s.	−0.490	n.s.
Lateral velocity	−0.557; 0.006	−0.508; 0.013	−0.467; 0.025	−0.415; 0.049	−0.480; 0.020	−0.495; 0.016	−0.621; 0.002	−0.535; 0.009	−0.573; 0.004	−0.604; 0.002
Mean velocity	−0.472; 0.023	−0.414; 0.048	n.s.	n.s.	−0.428; 0.041	−0.416; 0.048	−0.562; 0.005	−0.462; 0.026	−0.527; 0.010	−0.539; 0.008
Flexion-extension acceleration	n.s.	n.s.	n.s.	n.s.	n.s.	n.s.	−0.427; 0.042	n.s.	n.s.	n.s.
Rotational acceleration	n.s.	n.s.	n.s.	n.s.	n.s.	n.s.	−0.442; 0.035	n.s.	n.s.	−0.417; 0.046
Lateral acceleration	−0.521; 0.011	−0.472; 0.023	−0.426; 0.042	n.s.	n.s.	−0.443; 0.034	−0.573; 0.004	−0.477; 0.021	−0.542; 0.008	−0.553; 0.006
Mean acceleration	−0.432; 0.039	n.s.	n.s.	n.s.	n.s.	n.s.	−0.485; 0.019	n.s.	−0.443; 0.034	−0.459; 0.028
Distance	−0.481; 0.020	−0.423; 0.044	n.s.	n.s.	−0.424; 0.044	−0.418; 0.047	−0.560; 0.005	−0.462; 0.026	−0.526; 0.010	−0.538; 0.008
Area	−0.578; 0.004	−0.504; 0.014	−0.461; 0.027	n.s.	−0.477; 0.021	−0.489; 0.018	−0.640; 0.001	−0.545; 0.007	−0.606; 0.002	−0.623; 0.001
Angle	−0.417; 0.048	−0.478; 0.021	−0.429; 0.041	−0.453; 0.030	−0.439; 0.036	−0.460; 0.027	−0.470; 0.024	−0.495; 0.016	−0.516; 0.016	−0.511; 0.016
A-dimension	−0.711; <0.001	−0.649; 0.001	−0.595; 0.003	−0.568; 0.005	−0.639; 0.001	−0.647; 0.001	−0.754; <0.001	−0.703; <0.001	−0.700; <0.001	−0.748; <0.001
B-dimension	n.s.	n.s.	n.s.	n.s.	n.s.	n.s.	−0.540; 0.008	−0.423; 0.044	−0.575; 0.004	−0.525; 0.010

Data are expressed as *r* correlation coefficient (*p*-value). Abbreviations: CP, cerebral palsy; GMFM−88, Gross Motor Functional Measure; n.s.: not significant; BMI indicates body mass index.

**Table 4:** Summary of the stepwise regression analyses to determine predictors of functional state (GMFM-88 and PEDI total scores) of CP individuals (*n* = 24).

	Predictor Variables	*B*	Standard Error B	95% CI	Β	t	*p*	R^2^ Adjusted
GMGM-88 total score	Step 1 Flexion-extension angle	−2.569	0.633	−3.885, −1.254	−0.663	−4.062	0.001	0.413
	Step 2							0.548
	Flexion-extension angle	−2.734	0.587	−3.959, −1.508	−0.706	−4.653	<0.001	
	Age	−3.428	1.573	−6.708, −0.147	−0.331	−2.180	0.041	
	Step 3							0.638
	Flexion-extension angle	−2.607	0.542	−3.741, −1.472	−0.673	−4.809	<0.001	
	Age	−8.634	2.791	−14.485, −2.800	−0.833	−3.096	0.006	
	Height	100.671	46.130	4.121, 197.222	0.591	2.182	0.042	
PEDI total score	Step 1							0.560
	Area	−1.963	0.380	−2.753, −1.173	−0.748	−5.170	<0.001	
	Step 2							0.653
	Area	−3.037	0.577	−4.242, −1.833	−1.158	−5.261	<0.001	
	Rotational acceleration	0.094	0.040	0.010, 0.178	0.511	2.322	0.031	
	Step 3							0.790
	Area	−2.747	0.508	−3.814, −1.681	−1.047	−5.412	<0.001	
	Rotational acceleration	0.255	0.068	0.111, 0.398	1.387	3.721	0.002	
	Age	−2.265	1.078	−4.530, –0.001	−0.229	−2.102	0.050	
	Step 4							0.833
	Area	−2.486	0.484	−3.502, −1.470	−0.947	−5.139	<0.001	
	Rotational acceleration	0.357	0.082	0.186, 0.529	1.946	4.373	<0.001	
	Age	−3.012	0.967	−5.043, −0.981	−0.304	−3.116	0.006	
	Distance	−3.837	1.158	−6.270, −1.405	−1.643	−3.314	0.004	

Abbreviations: CP, cerebral palsy; GMFM−88, Gross Motor Functional Measure; PEDI, Pediatric Evaluation of Disability Inventory.

**Table 5:** Test–retest reliability of the new test outcomes.

		Intra-Day Reliability			
Spatial Plane		Second Day Data (Standard Deviation)	ICC (95% CI)	SEM	MDC_90_
CP group (*n* = 24)					
Flexion-extension angle (°)		8.29 (6.30)	0.826 (0.579, 0.928)	3.13	7.25
Rotational angle (°)		8.50 (8.48)	0.918 (0.804, 0.966)	2.83	6.57
Lateral angle (°)		6.67 (6.16)	0.821 (0.566, 0.926)	2.58	5.97
Mean angle (°)		7.82 (6.22)	0.923 (0.817, 0.968)	1.94	4.51
Flexion-extension velocity (°/s)		9.81 (9.00)	0.921 (0.812, 0.967)	2.66	6.16
Rotational velocity (°/s)		11.71 (13.03)	0.916 (0.799, 0.965)	4.00	9.30
Lateral velocity (°/s)		8.67 (8.21)	0.889 (0.731, 0.954)	2.91	6.75
Mean velocity (°/s)		10.06 (9.88)	0.919 (0.805, 0.966)	2.99	6.93
Flexion-extension acceleration (°/s^2^)		146.09 (153.44)	0.914 (0.795, 0.964)	45.54	105.61
Rotational acceleration (°/s^2^)		138.06 (138.83)	0.882 (0.714, 0.951)	54.91	125.02
Lateral acceleration (°/s^2^)		141.31 (127.34)	0.854 (0.645, 0.939)	54.79	127.07
Mean acceleration (°/s^2^)		141.82 (137.58)	0.892 (0.739, 0.955)	49.23	114.19
Distance (°/s)		11.83 (11.20)	0.929 (0.829, 0.971)	3.32	7.71
Area (°^2^)		944.56 (1599.32)	0.901 (0.761, 0.959)	467.85	1085.09
Angle (°)		−2.48 (22.44)	0.595 (0.334, 0.618)	15.74	36.50
A-dimension (°)		13.11 (10.67)	0.770 (0.439, 0.905)	5.49	12.74
B-dimension (°)		14.35 (16.72)	0.941 (0.860, 0.976)	3.73	8.64
Control group (*n* = 24)					
Flexion-extension angle (°)		4.45 (4.49)	0.652 (0.388, 0.850)	2.18	5.04
Rotational angle (°)		2.45 (2.38)	0.894 (0.757, 0.954)	0.80	1.86
Lateral angle (°)		2.08 (1.64)	0.774 (0.486, 0.901)	0.95	2.21
Mean angle (°)		2.99 (2.43)	0.934 (0.849, 0.972)	0.59	1.38
Flexion-extension velocity (°/s)		4.13 (2.23)	0.704 (0.332, 0.870)	1.69	3.91
Rotational velocity (°/s)		3.22 (3.48)	0.839 (0.631, 0.930)	1.85	4.29
Lateral velocity (°/s)		4.02 (2.37)	0.656 (0.321, 0.850)	1.93	4.48
Mean velocity (°/s)		3.79 (2.49)	0.751 (0.437, 0.891)	1.76	4.08
Flexion-extension acceleration (°/s^2^)		98.09 (60.10)	0.637 (0.365, 0.843)	40.86	94.76
Rotational acceleration (°/s^2^)		77.84 (55.80)	0.578 (0.048, 0.816)	38.53	89.37
Lateral acceleration (°/s^2^)		105.27 (62.22)	0.495 (0.000, 0.780)	54.08	127.74
Mean acceleration (°/s^2^)		93.74 (56.87)	0.587 (0.263, 0.820)	42.38	98.29
Distance (°/s)		5.45 (2.86)	0.522 (0.000, 0.791))	2.67	6.19
Area (°^2^)		82.59 (214.80)	0.927 (0.831, 0.968)	56.89	131.96
Angle (°)		3.02 (20.41)	0.514 (0.000, 0.792)	14.71	34.13
A-dimension (°)		4.98 (3.58)	0.944 (0.871, 0.976)	0.82	1.89
B-dimension (°)		3.01 (3.98)	0.841 (0.637, 0.931)	1.81	4.20

Abbreviations: CP, cerebral palsy; IMU, inertial measurement unit; ICC, intraclass correlation coefficient; CI, confidence interval; SEM, standard error of measurement; MDC, minimum detectable change.

## Supplementary Materials

The following are available online at https://www.mdpi.com/2075-4418/10/9/661/s1. Figure S1: Receiver operating characteristic (ROC) curve of the ellipse variables to discriminate between cerebral palsy individuals and controls; Figure S2: Receiver operating characteristic (ROC) curve of the ellipse variables to discriminate between non-wheelchair users and wheelchair users.
