# Supplementary material for: Design, Validity, and Reliability of a New Test, Based on an Inertial Measurement Unit System, for Measuring Cervical Posture and Motor Control in Children with Cerebral Palsy
Source: Diagnostics (Basel). 2020 Sep 1;10(9):661. doi: 10.3390/diagnostics10090661 (PMC7555956; doi:10.3390/diagnostics10090661)
Supplement: Supplementary file 1 [file diagnostics-10-00661-s001.pdf]

diagnostics

MDPI

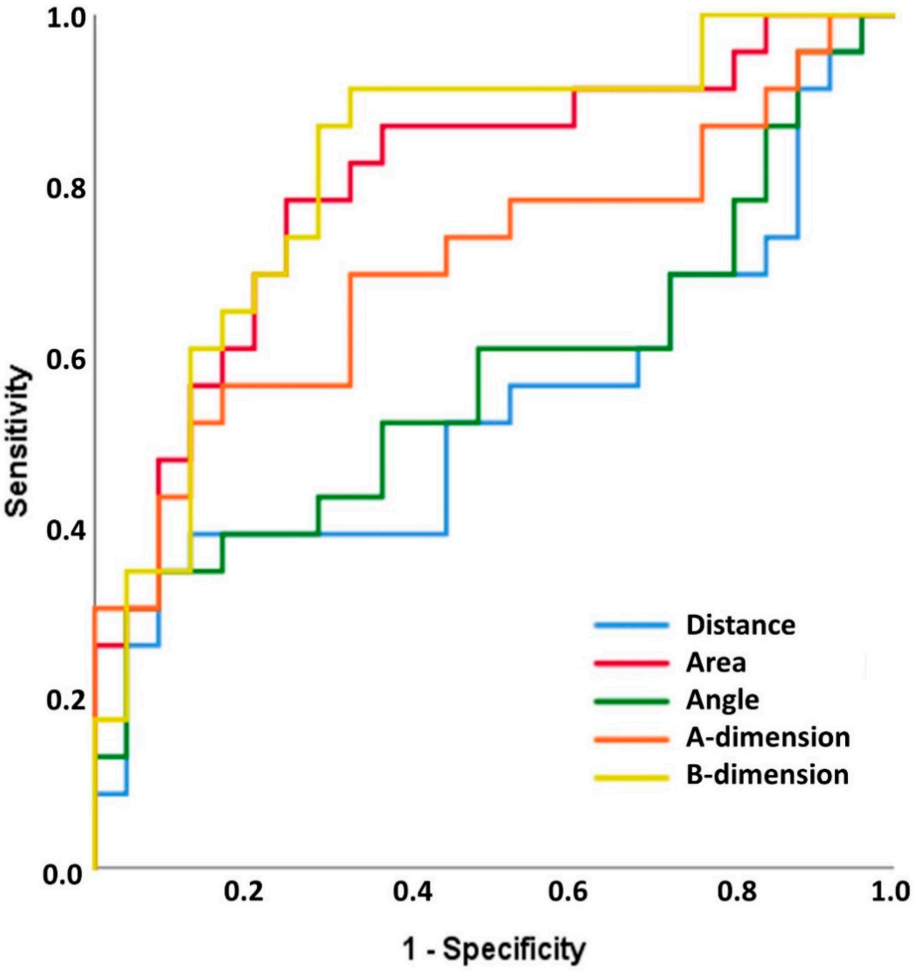

Figure S1. Receiver Operating Characteristic (ROC) curve of the ellipse variables to discriminate between cerebral palsy individuals and controls. The best cut-off points for the variables that showed statistical significance were as follows: for the area: 46.98°² (sensitivity: 78%; specificity: 76%; Area Under the Curve (AUC), 0.802, 95% CI = 0.674–0.929); for the B-dimension: 2.90° (sensitivity: 87%; specificity: 72%; AUC, 0.819, 95% CI = 0.697–0.942); for the A-dimension 4.59° (sensitivity: 70%; specificity: 68%; AUC, 0.708, 95%CI = 0.557–0.860).

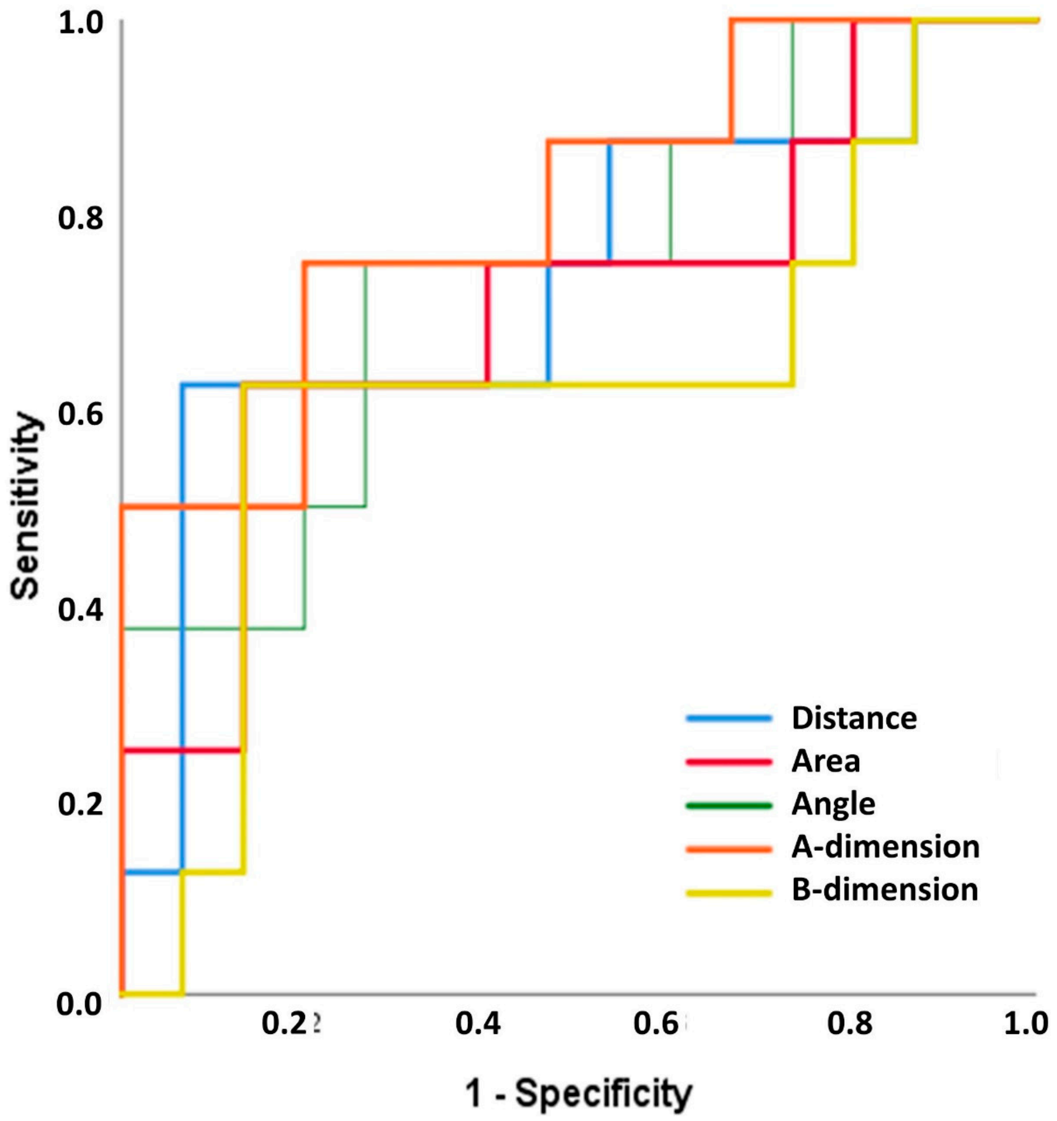

Figure S2. Receiver operating characteristic (ROC) curve of the ellipse variables to discriminate between non-wheelchair users and wheelchair users. The best cut-off points for the variables that showed statistical significance were as follows: for the area: 180.78°² (sensitivity: 75%; specificity: 66%; Area Under the Curve (AUC), 0.708, 95% CI = 0.502–0.948); for the A-dimension 11.50° (sensitivity: 75%; specificity: 80%; AUC, 0.808, 95% CI = 0.615–0.999).
